# Form and function: Optional complementizers reduce causal inferences

**DOI:** 10.5334/gjgl.134

**Published:** 2017-05-31

**Authors:** Hannah Rohde, Joseph Tyler, Katy Carlson

**Affiliations:** 1 University of Edinburgh, Edinburgh, UK; 2 Sense.ly, Oakland, CA, US; 3 Morehead State University, Morehead, KY, US

**Keywords:** discourse coherence, causality, complementizers, coordination, sentence comprehension

## Abstract

Many factors are known to influence the inference of the discourse coherence relationship between two sentences. Here, we examine the relationship between two conjoined embedded clauses in sentences like *The professor noted that the student teacher did not look confident and (that) the students were poorly behaved*. In two studies, we find that the presence of *that* before the second embedded clause in such sentences reduces the possibility of a forward causal relationship between the clauses, i.e., the inference that the student teacher’s confidence was what affected student behavior. Three further studies tested the possibility of a backward causal relationship between clauses in the same structure, and found that the complementizer’s presence aids that relationship, especially in a forced-choice paradigm. The empirical finding that a complementizer, a linguistic element associated primarily with structure rather than event-level semantics, can affect discourse coherence is novel and illustrates an interdependence between syntactic parsing and discourse parsing.

## 1 Introduction

Interpreting a sentence of natural language depends on the integration of a number of different levels of structure and meaning. A comprehender must understand the syntactic relationships that hold internal to that sentence as well as the semantic and pragmatic relationships that link that sentence to the larger discourse context. The inference of relationships between sentences or between propositions supports the establishment of overall discourse coherence, by which a comprehender makes sense out of the separate parts of a discourse.

In this paper, we focus on the link between markers of syntactic structure and comprehenders’ inference of cross-clausal coherence relations. First consider (1):

(1) A new mayor was elected. There was a riot.

The two sentences in (1) are structurally independent but could be understood to convey a forward cause-effect sequence whereby the mayor’s election preceded and caused the riot. In (1), such a relation could be left implicit or else marked with an explicit connective like *As a result*. A cause-effect sequence is just one example from a larger inventory of posited coherence relations, which include other causal relations (like the backward causal relation marked by *because*) as well as relations whose establishment depends on other types of reasoning, e.g. additive or contrastive (Mann & Thompson 1988; Polanyi 1988; Hobbs 1990; Sanders et al. 1992; Roberts 1996; Kehler 2002; Asher & Lascarides 2003; Prasad et al. 2008). Without an explicit connective between the two sentences, the inference of a connection between the two propositions depends largely on a comprehender’s world knowledge and, in the case of (1), on general reasoning about political elections and social unrest, along with a well-documented bias for inferring causal connections (Trabasso & van den Broek 1985; Louwerse 2001; Sanders 2005). Comprehenders can also rely on the presence of a variety of lexical cues to identify the speaker’s intended coherence relation: verb semantics, tense/aspect, negation, etc. (Kehler et al. 2008; Webber 2013; Asr & Demberg 2015; Dery & Koenig 2015). However, such cues typically contribute their own meaning and influence the sentence-level semantics and the discourse-level coherence relation in complex ways.

In the studies reported here, we examine effects on coherence from a lexical cue that contributes little semantic information to the sentence. Specifically, we test how the complementizer *that* influences coherence relations in embedded contexts like (2). These examples and the observation of the effect of the complementizer come from Bjorkman (2010, 2013).

(2)
  - The newspaper reported **that** a new mayor was elected and there was a riot.
  - The newspaper reported **that** a new mayor was elected and **that** there was a riot.

In (2a, b), the propositions about the mayor’s election and the occurrence of a riot are embedded under a verb which takes a sentence complement.^1^ As in (1), it is easy to interpret the election as the cause of the riot (a causal coherence relation), but it is also possible to interpret the two events as independent occurrences, which are mentioned together only because the newspaper reported both of them (what we will call the parallel or non-causal relation). The presence of the two complementizers in (2b) makes the syntactic relationship between the matrix and embedded clauses explicit, but the same embedding is supported even without the second complementizer, as in (2a). Bjorkman (2010, 2013) proposes that a non-causal interpretation is only available in contexts with a second overt complementizer, as in (2b), whereas the causal interpretation arises easily without the overt complementizer, as in (2a). The complementizer is a grammatical cue whose impact on sentence meaning can be said to arise indirectly via the embedded structure it signals at the syntactic level—or, at the semantic level, the subordinate dependency it creates between the embedded clause and its embedding verb (Portner 1992, 1997). Portner proposes that the role of *that* is to make the embedded proposition dependent for its interpretation on the matrix clause, such that the embedded proposition can receive its modal force via combination with the embedding verb. However, the complementizer itself contributes little to the propositional content, i.e., the event-level semantics in a comprehender’s mental model of who did what to whom. This is apparent in the optionality of the complementizer: *John reported (that) Mary left* indicates the same event in either form.

As such, what is intriguing about Bjorkman’s observation is that it highlights a small grammatical cue that may have the capacity to guide coherence establishment. The form of the sentence (the presence or absence of the overt complementizer) has the potential to determine the discourse-level meaning (a causal or non-causal coherence relation). It is also intriguing because the cue stands to undermine the well-known preference for causal inference. The studies reported here experimentally test Bjorkman’s claim and explore the possibility of gradience in participant judgments. Does the presence or absence of the second complementizer in passages like (2) influence comprehenders’ inference of a causal connection between the embedded propositions? Are non-causal interpretations available in constructions with and without the complementizer, or are they restricted to those with the second overt complementizer present?

The embedded structures that we target in this paper are not typical contexts for the study of coherence establishment, a domain which usually focuses on intersentential relations. However, there is a growing literature investigating the way that subsentential elements participate in coherence relations: prior work has identified contexts in which coherence relations hold between a matrix clause and a relative clause (H. Rohde et al. 2011; Kehler & Cohen submitted), a free adjunct (Stump 1985; Kortmann 1991; Reid 2015), or even an adjective (Webber 1991). When coherence relations operate within the sentence, this creates an opportunity for structural factors to interact with coherence relations in a way that is not possible in cross-sentence coherence relations. The studies reported here therefore draw attention to the interdependence of syntactic parsing and discourse parsing.

In the next section, we review literature on causal inference as a default, on known lexical cues that guide coherence establishment, and lastly on the role of the optional complementizer *that* in sentence processing. The paper then presents a series of studies using a variety of different probes. We test how the interpretation of a causal relation between two embedded clauses is affected by the presence or absence of the optional complementizer. When the second embedded clause is introduced by a complementizer, we find the predicted decrease in measures of forward causal inference. In addition, we test for the availability of a backward causal interpretation and find that it improves with the presence of the complementizer. Our results go beyond Bjorkman’s proposal and existing work on subsentential coherence relations by using experimental evidence to demonstrate that a lexical element which provides limited semantic information can have repercussions for pragmatic inference.

## 2 Background

### 2.1 Causal inference

Reasoning about relationships between events is fundamental to the ability to interpret not just text, but the world around us. The ability to infer causal connections underlies human understanding of physical systems (e.g., Shultz 1982), others’ mental states (e.g., Perner 1991), and essential but unseen properties of objects (e.g., Gelman & Wellman 1991). Within psycholinguistics, strong claims have been made regarding the centrality of causal relations to discourse interpretation: e.g., Trabasso and van den Broek’s (1985) emphasis on explanatory inferences in text understanding and Sanders’ (2005) causality-by-default hypothesis.

Comprehenders have been shown to favor causal connections in text, and such connections facilitate comprehension and recall (Trabasso & Sperry 1985; Trabasso & van den Broek 1985). Sanders argues that causal connections are processed more easily (Mak & Sanders 2012) and serve as an interpretational default (Sanders 2005), marshaling evidence that causal interpretations are assigned faster than non-causal alternatives (Sanders & Noordman 2000; Louwerse 2001), even though they are acquired later (Bloom et al. 1980; Evers-Vermeul 2005). Added evidence for this view comes from the relative preponderance of causal relations produced in a discourse continuation task (Tyler & Carlson 2015) and from the frequency of such relations in a large corpus of newspaper text with hand-annotated coherence relations (the Penn Discourse Treebank, PDTB; Prasad et al. 2008). Notably, the PDTB reveals that explicit connectives are rare for causal relations, which may reflect language users’ preference to treat such relations as the default when producing and interpreting juxtaposed sentences (Asr & Demberg 2012). In the PDTB, implicitness is therefore associated with causal inference, at least for the omission of connectives between sentences. In the conjoined embedded clauses at issue here, it is again implicitness that is associated with causal inference, though what is omitted is a complementizer, not a connective.

Most relevant for the current studies, a number of researchers have noted the ease with which the conjunction *and* invites a causal interpretation when it is used to conjoin two propositions (see reviews in Txurruka 2003; Zeevat & Jasinskaja 2007). Bar-Lev and Palacas (1980) make a strong claim that the lexical semantics of *and* itself imposes a basic constraint on the temporal/causal ordering of the conjuncts such that the second conjunct cannot occur prior to the first. This approach contrasts with more traditional analyses (Grice 1975; Schmerling 1975; Gazdar 1979; Posner 1980) in which *and* has an underlying symmetric meaning which undergoes pragmatic enrichment to achieve temporal/causal interpretations via the context of use and inferences about cooperative communication. In a similar vein, Txurruka (2003) pins the interpretive effects of *and* on its role in enforcing a coordinated discourse structure, distinct from the subordinated structures that are possible with sentence juxtaposition.

What is important for our purposes is the recognition of the preference for causal coherence as a backdrop against which to test our manipulations of the presence of a complementizer. Given the strength of causal biases, it is all the more intriguing how a cue as small as *that* could detract from a default causal interpretation.

### 2.2 Cues to coherence

Our studies’ manipulation of a lexical cue to guide coherence establishment fits within a larger literature that seeks to understand the contextual properties of a discourse that support the inference of particular coherence relations. Coherence relations can of course be signaled explicitly with an overt connective (e.g., *because, as a result, similarly, in contrast*, etc.), though many contexts leave the relation implicit. Moreover, the presence of an overt connective is not necessarily sufficient for disambiguation since a number of connectives are compatible with more than one relation. A conjunction like *and* can convey a simple additive reading (e.g., *Mary likes coffee and she likes tea*), but it also is compatible with the temporal and causal readings discussed above, as well as with contrastive readings (e.g., *Mary likes cats and John likes dogs*). In such cases, the content of the conjoined sentences is crucial for inferring the speaker’s intended relation, and this inferencing depends on contextual cues and comprehenders’ real-world knowledge.

Previous work has identified a number of cues that guide the inference of particular coherence relations when overt connectives are absent or ambiguous. Within the experimental pragmatics literature, story continuation tasks have been used to assess how cues in one sentence influence the relation which holds between that sentence and a subsequent sentence that a participant produces. There is evidence that both the type of event described in a context sentence and the way that event is described can influence the distribution of coherence relations in participant responses. For example, a preference for continuations that provide a reason (i.e., a backward causal relation) has been observed following sentences with Implicit Causality (IC) verbs, suggesting that the cause of an IC scenario, if unmentioned in prior discourse, is expected to be made explicit in a subsequent sentence (Simner & Pickering 2005; Kehler et al. 2008). Compare the story-continuation prompts in (3) and (4).

(3) John congratulated Emma. ___________________________
(4) John babysat Emma. ___________________________

Continuations following (3), which contains the IC verb *congratulate*, are more likely to address the question “Why” than continuations following (4), which contains the non-IC verb *babysit*. Events of congratulation typically have as their cause some praiseworthy action on the part of the congratulatee, whereas babysitting events do not appear to encode an implicit cause that merits mention to the same degree. In this way, the event type influences the identification of a discourse relation.

More subtly, aspectual marking on the verb itself can shift the distribution of coherence relations in participant story continuations. Events described as completed (perfective aspect) are shown to favor narrative continuations that tell what happened next, whereas events described as ongoing (imperfective aspect) favor continuations that explain or elaborate on the event (Kehler et al. 2008; see also Smith 1991 on the distinction between the use of perfective and imperfective in narrative text). In a similar vein, Dery and Koenig (2015) report that context sentences that represent an event as temporary yield a preference for continuations that move the discourse in time (forward or backward) more than context sentences representing an event as permanent. Even the subsequent mention of a particular event participant in the prompt can bias the types of continuations participants produce, for example disfavoring causal continuations following an IC verb like *congratulate* if an event participant other than the causally implicated congratulatee is mentioned (Kehler & H. Rohde 2017).

Beyond data from behavioral studies, the PDTB offers a large-scale resource for analyzing the distribution of coherence relations and the surface properties that co-occur with those relations. For example, computational linguists have tested whether certain features occur more frequently when particular relations hold: adverbials like *Monday* or *yesterday* in temporal relations, lexical items conveying opposite polarity sentiment in relations of comparison (*John is* ***terrible*** *whereas Mary is* ***wonderful***, Pitler et al. 2009), and negation in relations related to contrast and alternatives (Webber 2013; Asr & Demberg 2015).

As a last example, information structural marking can provide another cue that may be used for inferring coherence relations. Kehler (2005) argues that different patterns of focus marking are associated with different relations in contexts like the one shown in (5). In (5), the connective *and* is ambiguous between at least two readings. The two readings differ in what larger discourse question the passage is taken to answer—*Who did what to whom?* (parallel) or *What happened and what was the consequence?* (result).

(5) Powell defied Cheney, and Bush punished Powell.

Kehler notes that the parallel interpretation is associated with accent placement on each word of the second clause (*BUSH PUNISHED POWELL*), whereas the result interpretation leaves the final word unaccented (*BUSH PUNISHED Powell*). Focus marking, as indicated by prosodic accenting (represented here by the use of all caps), thus influences the inferred relation or question under discussion (Roberts 1996; Büring 2004). The manipulation of surface form through focus marking has been shown to influence the interpretation of other pragmatic phenomena such as coreference, implicature, and projection (Cummins & H. Rohde 2015; Simons et al. to appear).

In these cases, however, the cues themselves convey information that contributes to the event-level semantics of who did what to whom (verb class, verb aspect, reference, negation, focus). The studies presented here crucially manipulate the optional complementizer that can appear at the beginning of an embedded clause, a cue that itself carries primarily information about structure building and propositional mood (the latter in combination with the lexical semantics of the embedding verb).

### 2.3 Interpretation of "and" and its sensitivity to the complementizer "that"

The conjunction *and* is often ambiguous, as can be seen in (5) above and here in (6), in which the two sentences in (1) are conjoined.

(6) A new mayor was elected and there was a riot.

The two events described in (6) can be interpreted as two unrelated events in a parallel or symmetric relation, in which case conjunct reversal is permitted. Under the causal interpretation, the two events are connected asymmetrically and the order of the conjuncts cannot be reversed. Asymmetric readings of *and* arise easily and must be accounted for, as Gazdar (1979: 168) highlighted with his famous, now rather dated, example (*Getting married and having a child is better than having a child and getting married*; originally from Wilson 1975: 151, with similar examples discussed in Cohen 1971). Such readings are typically treated as pragmatic inferences (Grice 1975; Schmerling 1975; Posner 1980; Carston 1993). Clauses conjoined with *and* are said to permit the inference of fewer possible relations than two independent sentences that are juxtaposed with no connective (e.g., Bar Lev & Palacas 1980). This means that there are only a few possible relations under consideration in the conjoined contexts of interest here. For our purposes, the relevant ones are those in which (i) the event described in the first clause caused the event described in the second, and hence followed the first temporally (forward causality) or (ii) the two events are unordered temporally and stand in no causal relation (parallel). In addition, we will consider an alternative causal interpretation whereby the event described in a second clause is not the result but the explanation of the first (backward causality, see Carston 1993; Wilson & Sperber 1998).

Conjoined clauses can be embedded, as in (2), repeated here as (7). The presence of the complementizer *that* is optional, and its presence or absence before the second conjunct appears to influence the interpretation of the relation that holds between the two embedded clauses.

(7)
  - Th**e** newspaper reported **that** a new mayor was elected and there was a riot.
  - The newspaper reported **that** a new mayor was elected and **that** there was a riot.

Bjorkman provides a syntactic analysis for why the presence of an additional complementizer (as in (7b)) would favor a non-causal interpretation and its absence (as in (7a)) would favor a (forward) causal interpretation. In her terms, the embedded clauses in (7a) are ambiguous between complementizer phrase (CP) and tense phrase (TP) coordination whereas (7b) is unambiguously CP coordination, the more complex and larger syntactic structure. See (8) for a bracketed illustration of the syntactic difference, with (8a) indicating the more economical conjoined TP structure that is possible in the absence of the second complementizer. However, the more complex structure in (8b) can also be built even when the complementizer is not present.

(8)
  - The newspaper reported [_CP_ that [_TP_ the mayor was elected] and [_TP_ there was a riot]].
  - The newspaper reported [_CP_ that [_TP_ the mayor was elected]] and [_CP_ (that) [_TP_ there was a riot]].

Bjorkman states that “asymmetric [causal] interpretations are available only to TP coordination, while logical [non-causal] interpretations are available only to CP coordination, at least in embedded contexts” (2010: 12–13). She presents cross-linguistic data indicating a correlation between the presence/absence of the complementizer and the availability of a causal interpretation. In a psycholinguistic follow-up to Bjorkman’s analysis of CP versus TP coordination, Thompson, Collado-Isasi, Omana and Yousuf (2012) measured processing difficulty associated with logical, temporal, and causal interpretations of *and* in non-embedded contexts. They had participants read a sentence one word at a time and then timed participants’ subsequent production of that same sentence. Sentences conveying logical conjunction (*Gabriel ordered the pasta and Lily had some chicken*) yielded longer production times compared to sentences conveying temporal or causal meanings (*She won the lottery and they bought a yacht; A hurricane hit and the schools closed*). They take these findings as support for Bjorkman’s claim that logical conjunction requires the more complex CP structure, whereas asymmetric interpretations use the less complex TP structure. The results are also in keeping with the generalization reviewed earlier that causal interpretation facilitates processing.

There is a separate line of research on optional complementizers in the sentence processing literature, which focuses on simpler structures consisting of a single complement clause or a relative clause (e.g., Ferreira & Dell 2000; Roland, Elman & Ferreira 2006; Torres Cacoullos & Walker 2009; Jaeger 2010; Wasow, Jaeger & Orr 2011). Although that work explores biases in the production of *that* rather than its interpretive effects, the findings nevertheless can help contextualize our studies. That prior work assessed local syntactic and semantic factors that predict *that* omission in single embedded complement clauses (e.g., *I think (that) you must go*). In a corpus analysis of optional *that*, Jaeger (2010) compares multiple factors that are posited to predict a speaker’s choice to include or omit the optional complementizer. He finds that the most influential predictor is the information density of the material at the onset of the verb’s complement. For example, *that* is more likely to be present if the embedding verb does not typically take a complement clause, meaning that the presence of the complementizer may be used in part to herald an atypical structure. The verb *worry*, which rarely takes a complement clause, had the highest *that*-bias; the verb *guess*, which nearly always takes a complement clause, had the lowest *that*-bias. In other words, when the syntactic structure is the default for a particular verb, omission of *that* increases in production. In the studies reported here we ask whether *that* omission is associated with the inference of a default coherence relation, though in our cases, the complementizer that is at stake is the one that can appear on the second of two conjoined embedded clauses.

In sum, the inference of causal connections has been argued to function as a common default in language processing. Causal coherence relations can be lexically cued, and when they are not, recourse is made to world knowledge and the content of clauses to explain perceptions of causal coherence. This paper explores whether a new source of information, the surface cue of an optional complementizer before the second of two conjoined embedded clauses, may affect interpretations of causal coherence, and explores how such effects can be explained. We specifically test whether psycholinguistic judgments of meaning and felicity are sensitive to this interaction between surface form and discourse coherence. This work is an opportunity to test whether Bjorkman’s predictions, which make only limited allowance for gradience, are upheld in participant judgments.

## 3 Experiment 1 (rating scale)

To test the role of the complementizer on causal inference, this first study presented participants with sentences containing two embedded clauses, with or without an optional complementizer before the second of the two clauses (analogous to (6) and (7)). Participants’ ratings of the causal connection between the two clauses allow us to test whether the presence of *that* blocks the causal interpretaton.

### 3.1 Method

#### 3.1.1 Materials

The study consisted of 32 target sentences along with 20 fillers. A sample item is shown in (9) (for all target sentences, see Appendix A).

(9)
  - During the class observation, the professor noted that the student teacher did not look confident and the students were poorly behaved. [COMPLEMENTIZER ABSENT]
  - During the class observation, the professor noted that the student teacher did not look confident and that the students were poorly behaved. [COMPLEMENTIZER PRESENT]

Target sentences used a variety of embedding verbs (e.g., *announced*, *claimed*, *remarked*, *feared*, etc.). The two events described in the embedded clauses were selected to allow for the possibility that the first could reasonably have caused the second but need not have. We attempted to avoid pairs of events whose causal link would already be at ceiling given world knowledge, with the construction of the items being based on our own intuitions about the events. We manipulated the presence/absence of a complementizer *that* before the second embedded clause. Most items began with a prepositional phrase that was designed to give more context and to improve the plausibility of a non-causal reading: for example, in (9), class observations are taken to be situations that could plausibly yield a list of non-causally-related comments about the classroom.

Of the 20 fillers, 10 were unambiguous catch trials, such that a participant’s poor performance on them would indicate lack of attention or a misunderstanding of the task. Causality was manipulated within these 10 fillers such that each participant saw 5 in the unambiguously causal condition (marked with the discourse marker *because*) and 5 in the unambiguously non-causal condition (marked with *despite* or *unrelated to*; see Appendix C), as in (10).

(10)
  - At the community swimming pool, the lifeguard Kyle reprimanded Sally because she was biting someone. [causal]
  - At the community swimming pool, the lifeguard Kyle reprimanded Sally unrelated to her biting someone. [non-causal]

A further 10 fillers manipulated the IC/non-IC status of the verb (see Appendix D) and were included to test whether participants would indeed favor a causal interpretation in IC contexts, as has been shown in previous work, and furthermore whether they would do so even when an existing causal connection could be inferred to hold between subsentential elements. For example, in (11), a causal connection may hold between Sheila’s lecture and the intern’s texting (see Webber 1991; Kehler & H. Rohde 2015; Kehler & Cohen submitted). Verb class was manipulated within items for these 10 fillers; each participant saw 5 IC and 5 non-IC sentences.

(11)
  - During a business conference, the manager Sheila lectured the texting intern Kevin. [IC]
  - During a business conference, the manager Sheila introduced the texting intern Kevin. [non-IC]

#### 3.1.2 Participants

Forty participants, recruited through Amazon Mechanical Turk, completed the experiment through a survey on Qualtrics. Two participants were excluded from the analysis for assigning higher ratings of causal connection to the unambiguously non-causal filler sentences than the unambiguously causal filler sentences. One non-native speaker of English was also excluded. The results represent the data from the remaining 37 participants. The research was registered with the University of Edinburgh’s Linguistics and English Language ethics review board.

#### 3.1.3 Procedure

Before starting the survey, participants were familiarized with the type of sentences they would be rating with a practice sentence like (12a) or (12b). Whether participants saw a practice sentence with or without the complementizer was randomized. While not discussing the optionality of the complementizer, the instructions did note the possible causal connection between the two embedded clauses (yelling could lead to a missed free throw but need not).

(12)
  - At the basketball game, the referee observed that a fan screamed from the bleachers and the star player missed his free throw. [COMPLEMENTIZER ABSENT]
  - At the basketball game, the referee observed that a fan screamed from the bleachers and that the star player missed his free throw. [COMPLEMENTIZER PRESENT]

This familiarization was intended to help reduce competition from the alternative interpretation in the complementizer-absent condition whereby only one clause is embedded and the second is an independent clause (i.e., for (12a), the referee observed the yelling and then subsequently the star player missed the free throw). In that structure, the conjunction *and* in (12a) joins the statement about the referee’s observation with the statement about the star player’s missed throw, two events that are less likely to be causally linked than the yelling and the missed throw. If participants frequently assign the alternative structure, this would serve only to reduce our ability to see the predicted effect: the complementizer-absent condition is predicted to yield higher causal ratings, but if participants favor the alternative structure when the complementizer is absent, their causal ratings would likely go down.

The 32 target sentences and 20 fillers were presented in a random order. After each sentence, participants were asked to rate the causal connection between two parts of the sentence on a 1–5 scale (from definitely not causally related to definitely causally related). For a target sentence like (9), they would be asked how likely it is that the students’ poor behavior was caused by the student teacher not looking confident. For a catch trial like (10), they would be asked how likely it is that the lifeguard reprimanded Sally because of her biting. For an IC filler sentence like (11), they would be asked how likely it is that Sheila’s lecture was caused by Kevin’s texting (or in the non-IC condition, how likely it is that Sheila’s introduction was caused by Kevin’s texting). Each sentence and question pair appeared on a screen by itself, and participants had to choose a rating to advance to the next item.

### 3.2 Results and discussion

Participants’ ratings were modeled with a cumulative linear mixed model using the clmm function in the ordinal package (Christensen 2015) in R (R Development Core Team 2015). The model of the target items contained a fixed effect for complementizer presence and random intercepts and slopes by participant and item, the maximal random effects structure (Barr et al. 2013). Model comparison was used to test for a difference in the likelihood of the data under a model with or without the fixed effect of complementizer. Analyses of the filler items were conducted similarly, substituting a fixed effect of filler type for complementizer.

The ratings on the unambiguous filler items confirm that the 37 participants we included in the main analysis paid attention to the task and understood the direction of the rating scale: unambiguously causal fillers received higher causal ratings (4.70) than unambiguously non-causal fillers (2.55), and model comparison confirmed that causality ratings were better captured by a model containing a factor for discourse marker *because*-vs-*despite* (ß = −5.17, p < 0.001). In the subset of the fillers in which we varied verb type, the presence of an IC verb yielded higher causal ratings (4.14) than a non-IC verb (2.61); model comparison confirmed that verb class was a significant predictor of ratings (ß = −2.98, p < 0.001). This suggests that participants were paying attention to causality in the expected manner.

The data for the target items consists of 1,184 judgments (no questions skipped; all participants completed the task). In accordance with Bjorkman’s predictions that an optional complementizer can block causal interpretations, sentences with the optional complementizer present received lower mean causality scores (3.08) than sentences without the optional complementizer (3.21). Complementizer presence was found to be a significant predictor of causality ratings (ß = 0.13, p < 0.05).

Given the subtlety of the rating difference between the two conditions, Experiment 2 used a different methodology (a forced-choice task) to test how highlighting the presence/absence of the complementizer may influence causal interpretation. It has been argued that rating tasks are most appropriate when effect size is what is of interest, whereas forced-choice tasks are better at detecting qualitative differences between conditions (Schütze & Sprouse 2014). Our tested prediction can best be understood as a qualitative distinction: for a given target sentence, the participant must decide whether the two described events are causally linked or whether they occurred independently. Participants’ estimates of which scenario is at play may be graded, but the two scenarios themselves are fully distinct; the two events in the world cannot have both a causal relationship and an independent relationship since the two are mutually exclusive. Experiment 2 draws attention to the two real-world scenarios and two linguistic formulations that could be used to describe such scenarios.

## 4 Experiment 2 (forced choice)

This study again contained target sentences in which the descriptions of two events appear as conjoined embedded clauses. However, in this study participants were presented with both the complementizer-absent and complementizer-present variants of each target item and asked to choose which variant conveys a specific meaning. The predictions, based on Bjorkman’s claims and the results of Experiment 1, are that participants will select the variant without the complementizer when asked which version conveys a causal relationship between events and will select the variant with the complementizer when asked which version conveys that the events are unrelated.

### 4.1 Method

#### 4.1.1 Materials

This study consisted of 28 target sentences (see Appendix B). The target items were adapted from Experiment 1, with the elimination of partially overlapping sentences and the standardization of the presence of an initial prepositional phrase across all sentences. Each target item was presented as a pair of sentences varying only in the presence/absence of the complementizer. The 10 unambiguous catch trials were also adapted from Experiment 1, with the non-causal condition edited to use only the discourse marker *unrelated to*. Each catch trial was presented as a pair of sentences: the unambiguously causal condition (with *because*) and the unambiguously non-causal condition (with *unrelated to*). The 10 IC fillers were adapted from Experiment 1, with the non-IC verbs eliminated and with the modifier appearing either as a prenominal adjective (*the texting intern*, as in Experiment 1) or as a relative clause (*the intern who was texting*) to test whether an integrated pre-nominal adjective is perceived as more causally linked than the post-nominal clause. Each IC filler trial was presented as a pair of sentences: the variant with the post-nominal relative clause and the variant with the pre-nominal adjective.

#### 4.1.2 Participants

Forty-two participants, recruited through Mechanical Turk, completed the experiment through a survey on Qualtrics. All participants were native speakers of English. Five participants were excluded from the analysis for more than one incorrect answer on the unambiguous filler sentences. The results are from the remaining 37 participants. The research was approved by the Morehead State University Institutional Review Board for the Protection of Human Subjects (IRB), protocol 11-09-12R7.

#### 4.1.3 Procedure

Before starting the survey, participants were shown the paired examples in (12) and the causal and non-causal interpretations were noted. They were then told they would be asked to select which version they preferred in response to a question about the sentence meaning, as in (13). Whether they saw a practice question about causality or non-causality was randomized.

(13)
  - Which of the two sentences is more likely to mean that the fan screaming from the bleachers caused the star player to miss his free throw? [causal question]
  - Which of the two sentences is more likely to mean that the fan screaming from the bleachers and the star player missing his free throw were unrelated? [non-causal question]

For the main experiment, each item was presented as a pair of sentences with a question. Whether participants were asked about causality or unrelatedness on a given item was counterbalanced within-participants, and the order of the 48 questions was randomized for each participant.

### 4.2 Results and discussion

We modeled the binary choice of sentence selection with a logistic mixed effects model using the lmer function in the lme4 package (Bates, Maechler & Bolker 2013) in R (R Development Core Team 2015). The model of the target items contained a fixed effect for question type and random intercepts and slopes by participant and item, the maximal random effects structure (Barr et al. 2013). Model comparison was used to test for a difference in the likelihood of the data under a model with or without the fixed effect of question type. Analyses of the filler items were conducted similarly, substituting a fixed effect of filler type for question type.

As in Experiment 1, the catch trials confirm that the 37 participants we include in the main analysis paid attention to the task and understood the questions: the unambiguously causal variant with *because* was chosen when the question asked about one event causing the other (100% of the time); the unambiguously non-causal variant with *unrelated to* was chosen when the question asked about unrelated events (99.5% of the time). For the IC fillers, the variant with pre-nominal modification was selected more often (65.4% of the time) when the question asked about one event causing the other; the variant with the post-nominal relative clause was selected more often (64.9% of the time) when the question asked about unrelated events. Results show that question type is a significant predictor of preferred modification position (ß = 1.77, p < 0.005).

The data for the target items consists of the full 1,036 responses. The results showed a strong effect of the complementizer on causal interpretation, whereby question type was found to be a significant predictor of preferred sentence (ß = 4.40, p < 0.001): in response to questions asking participants to select the sentence that is more likely to contain causally related propositions, participants preferred the sentence without the second complementizer 79% of the time. Conversely, in response to questions asking for the sentence that is more likely to contain unrelated propositions, participants preferred the sentence with the second complementizer present 80% of the time. See Table 1.

Experiments 1 and 2 provide evidence that an optional complementizer reduces the perception of causal coherence between embedded clauses and does so especially strongly with a forced-choice methodology. The target sentences used in both studies all contained pairs of embedded clauses designed to be ambiguous between causal and non-causal interpretations. In cases of *and*-conjunction, causal inferences (almost) always involve the first clause describing the cause of the second: a forward causal relationship. Txurruka (2003) gives an account in which *and* permits coordinating discourse relations but not subordinating relations like that conveyed by *because*. However, an alternative reverse causal relationship is occasionally possible whereby the second clause describes the cause of the first, as shown in the following example from Larry Horn (cited in Carston 1993).

(14)
  - Did John break the glass?
  - Well, the glass broke and John dropped it.

If optional complementizers really do reduce causal interpretation, then an additional prediction is that clauses whose propositional content suggests a reverse causal relationship should be more acceptable with the extra complementizer present than without it. That is, if the default interpretation of *and*-conjoined clauses is to infer forward causality (where the first clause causes the second) and if the optional complementizer reduces this inference, then cases in which the inference of forward causality is highly implausible should be more acceptable with the optional complementizer (as also observed by Bjorkman 2010, 2013, and traced to the TP vs. CP coordination structure). In Experiment 3, we test the effect of a complementizer in contexts in which the propositional content of the conjoined clauses favors a reverse causal relationship.

## 5 Experiment 3: Reverse causality (rating scale)

This study measures the impact of the presence or absence of an optional complementizer on the acceptability of sentences with two embedded clauses, where the propositional content suggests, or even requires, that the second caused the first. In (15), a forward causality result relation is easily inferred to hold between the two embedded clauses (running a red light can easily lead to being pulled over). The reverse causality case in (16) sounds relatively degraded because people rarely get caught by the police before they do something illegal.^2^

(15) The witness said that Clive ran a red light and the policeman pulled Clive over.
(16) The witness said that the policeman pulled Clive over and Clive ran a red light.

Conjunction with *and* is often interpreted as conveying that the described events are moving forward in time. In the case of (16), the propositional content is incompatible with such an interpretation (or at least requires more imagination to construct a relevant context). If there were a way (e.g., an optional complementizer) to reduce the inclination to infer forward causality in (16), then the sentence is predicted to sound better. Sentences (17) and (18) include the complementizer.

(17) The witness said that Clive ran a red light and that the policeman pulled Clive over.
(18) The witness said that the policeman pulled Clive over and that Clive ran a red light.

Sentence (17) is predicted to sound acceptable if the additional complementizer does not fully block the forward causality. Sentence (18) is predicted to sound better than (16) if the optional complementizer, as suggested by Experiments 1 and 2, reduces perceptions of causality. The optional complementizer in (18) should loosen the expectation of a forward causal interpretation, thereby opening up alternative interpretations (e.g., logical conjunction, reverse causality) and improving acceptability. The effect is predicted to emerge as an interaction between order and complementizer. Paralleling the forward causality studies, we first report the rating study and then describe a forced-choice variant.

### 5.1 Method

#### 5.1.1 Materials

There were 20 target sentences of the types shown in (15–18), varying the order of the two embedded clauses and the presence of a complementizer before the second embedded clause (see Appendix E). The survey contained 80 filler sentences, including 44 items for two unrelated experiments. The interleaved experiments contained clefts that varied in acceptability and the grammatical case of post-copula pronouns (*It was I who completed the assignment* vs. *It was me who completed the assignment* vs. *It was me whom completed the assignment*) and sentences with focus-sensitive particles *even* and *only*. The remaining 26 fillers used a variety of structures, including several catch trials that were fully ungrammatical in order to identify participants who were not paying attention or misunderstood the task.

#### 5.1.2 Participants

125 participants were recruited from two universities. Seven were students at Morehead State University who responded to a posting for a paid study on a website listing available experiments for students in psychology classes; they received $10 for their participation. The remaining participants were students at South Georgia State College taking introductory English classes who participated in exchange for extra credit.^3^ The research was approved by the South Georgia State College Institutional Review Board and by the Morehead State University IRB (protocol 11-09-12R8). Nine participants were excluded because they rated at least two ungrammatical filler sentences highly (with a 6 or 7 for sentences such as *It was the softball who broke the window). The results are from the remaining 116 participants. All were native speakers of English.

#### 5.1.3 Procedure

Participants were asked to rate on a 1–7 rating scale how natural and acceptable each sentence was, where 1 indicated unnatural/unacceptable and 7 indicated natural and acceptable. Sentences were counterbalanced for the presence/absence of the second optional complementizer.

### 5.2 Results and discussion

The analysis here follows the ordinal modeling described in Experiment 1 with fixed effects for causal order (forward/reverse), sentence type (with/without the complementizer), and their interaction, along with random intercepts and slopes by participant and item, the maximal random effects structure. The fixed effects were centered via deviation coding to ensure that the main effects would be interpretable with the interaction present.

The data from the 116 participants consists of 2,306 ratings (14 cells were empty because the participant did not answer the question). Table 2 shows the means for the four conditions. The only significant effect is for sentence type: forward causality sentences were rated higher than reverse causality sentences (ß = −1.11, p < 0.001). Aside from that, the presence of the complementizer yielded numerically higher ratings (ß = 0.15, p = 0.11), with a slightly larger improvement due to the complementizer for reverse causality sentences in keeping with our predictions (p = 0.22).

These results show that participants prefer forward causality, but there is no significant evidence that the acceptability of sentences in the reverse causality condition improves in the presence of a complementizer. However, Experiment 1 and 2 showed that a small effect in a rating-scale measure was more apparent in a forced-choice paradigm. It is possible the same could be true for reverse causality sentences. Experiment 4a tested this, using a forced-choice methodology to assess the effect of an optional complementizer on the acceptability of reverse causality sentences.

## 6 Experiment 4a: Reverse causality (forced choice ratings)

This study presented participants with both the complementizer-absent and complementizer-present variants of each target item and asked them to select which variant sounds better. If complementizer presence favors reverse causality more than forward causality, participants’ selections are predicted to vary with the causal order. Given the numeric pattern of the Experiment 3 rating results, we predict a preference for sentences conveying forward causality and those that contain a complementizer.

### 6.1 Method

#### 6.1.1 Materials

Each item consisted of a question prompt followed by a pair of candidate sentences. For the 20 target items, the question prompt was *Which sentence sounds better to you?* The pair of candidate sentences differed in the presence/absence of the complementizer but kept causal order constant as in (19a–b) and (20a–b). The candidate sentences were the same as in Experiment 3 (see Appendix E).

(19) Forward-causality
  - The witness said that Clive ran a red light and the policeman pulled Clive over. [COMPLEMENTIZER ABSENT]
  - The witness said that Clive ran a red light and that the policeman pulled Clive over. [COMPLEMENTIZER PRESENT]
(20) Reverse-causality
  - The witness said that the policeman pulled Clive over and Clive ran a red light. [COMPLEMENTIZER ABSENT]
  - The witness said that the policeman pulled Clive over and that Clive ran a red light. [COMPLEMENTIZER PRESENT]

The study also contained 18 items for an unrelated experiment on ellipsis ambiguity (e.g. *Mika wanted to bake muffins before Leah did*). For those items, the prompt included the ambiguous sentence followed by the question *Which is true?* The pair of candidate sentences identified two possible interpretations (e.g., *Mika wanted to bake muffins before Leah wanted to bake muffins; Mika wanted to bake muffins before Leah baked muffins*). A further 35 fillers showed a sentence and asked an interpretation question (if the sentence was ambiguous) or a comprehension question (if the sentence was unambiguous), such as *Who did what?* or *What happened?*

#### 6.1.2 Participants

We recruited 31 participants through Amazon Mechanical Turk who completed the experiment through a survey on Qualtrics. All participants were native speakers of English and none were excluded. The research was approved by the Morehead State University IRB (protocol 11-09-12R8).

#### 6.1.3 Procedure

For each item, participants answered the prompt question by selecting one of the two candidate sentences. For target items, we counterbalanced causal order.

### 6.2 Results and discussion

The binary outcome of sentence choice was modeled using a logistic mixed-effects model with a fixed effect of causal order and random intercepts and slopes for participant and item (the maximal random effects structure).

The data from the 31 participants consisted of 620 responses. Table 3 shows the selected sentence type (absence/presence of *that*) for the forward and reverse causality orders.

As predicted, causal order was a significant predictor of sentence selection (ß = 2.05, p < 0.001): in the forward-causality condition, participants preferred the version with *that* 60% of the time, and, as predicted, in the reverse-causality condition that preference increased to 84%. It seems that when events constrain causal order so that the second clause is most easily interpreted as the cause of the first, there is a stronger preference for a sentence form that includes the optional complementizer. The presence of the complementizer biases away from a forward causal interpretation and the absence of the complementizer strengthens the preference for a forward causal interpretation.

## 7 Experiment 4b: Reverse causality (interpretation)

In Experiment 4a, we asked participants to rate sentences with forward or reverse causality orders, comparing items with and without *that*. However, we merely asked which sentences they preferred, not what interpretation they formed for the sentences. It is possible, therefore, that participants did not draw the conclusion of reverse causality, and instead may have accepted the embedded clauses as simply discussing two independent events that occurred. Because of that gap, we carried out an additional study using the reverse causality items from Experiment 4a and we explicitly probed the reverse causality meaning.

### 7.1 Method

#### 7.1.1 Materials

Each item consisted of a sentence followed by a question asking whether the reverse causality meaning was possible. The 20 target sentences were the reverse-causality sentences from Experiment 4a (see Appendix E) and appeared either with or without the second complementizer, as in (21a–b). The questions were yes/no questions asking if the second conjoined embedded clause could have caused the first, as in (21c). A positive response yes signaled that the participant endorsed the possibility of the reverse-causality interpretation.

(21) Reverse-causality
  - The witness said that the policeman pulled Clive over and Clive ran a red light. [COMPLEMENTIZER ABSENT]
  - The witness said that the policeman pulled Clive over and that Clive ran a red light. [COMPLEMENTIZER PRESENT]
  - Can this sentence express the meaning where Clive running a red light caused the policeman to pull him over? Yes/No.

In order to provide some variety and a point of comparison, 6 targeted fillers were created which contained embedded clauses only compatible with a forward causality relationship, as in (22a). Three of these contained two complementizers and three had only the first complementizer.

(22) Forward-causality fillers
  - The news anchor stated that the flooding was severe and that residents were advised to evacuate the area.
  - Can this sentence express the meaning where residents being advised to evacuate caused the severe flooding? Yes/No.

These fillers were also followed by questions asking if the second clause could have caused the first, as in (22b).

The study contained 18 items for an unrelated experiment on attachment ambiguity (e.g., *It’s the box on the bookcase with ivory inlay*). For those items, the prompt was a yes/no question about the high attachment or low attachment interpretation (*Does this refer to a box with ivory inlay?*). A set of 10–13 fillers, depending on the list, contained short sentences with *only* before the verb or the object (*The engineer (only) repaired (only) a machine*) and asked participants to rate the naturalness of the sentences on a 1–7 scale. A further 33–36 fillers showed a sentence and asked an interpretation question (if the sentence was ambiguous) or asked participants to complete the sentence (if it was incomplete), for a total of 90 items per list. There were two lists of items, pseudorandomized such that no consecutive items were of the same sub-type or condition.

#### 7.1.2 Participants

We recruited 45 participants through Amazon Mechanical Turk who completed the experiment through a survey on Qualtrics for a payment of $3.75. All participants reported being native speakers of English. The responses to 22 unambiguous filler questions were examined, and any participant with less than 90% accuracy on these items was eliminated. An additional participant was eliminated. An additional subject was removed due to responses that indicated acceptance of the 6 forward-causality filler sentences as conveying reverse causality. This left a total of 41 participants. The research was approved by the Morehead State University IRB (protocol 11-09-12R8).

#### 7.1.3 Procedure

For each item, participants answered the prompt question by choosing yes or no answers. For target items, we counterbalanced items with and without *that*, so that each participant saw an equal number of items in each condition and no items more than once.

### 7.2 Results and discussion

The reverse-causality sentences with *that* showed 73% positive answers (endorsing the reverse-causality interpretation), and sentences without *that* received 67% positive answers. The binary outcome of accepting a reverse-causality interpretation was modeled using a logistic mixed-effects model with a fixed effect of presence of *that* and random intercepts and slopes for participant and item (the maximal random effects structure). This analysis showed that the additional *that* significantly increased reverse-causal interpretations (ß = 0.64, p < 0.05).

In an additional analysis, we compared responses to the 20 reverse-causal items with the responses to the 6 forward-causal fillers. As expected, the forward-causal fillers received an average of only 20% positive answers to the reverse-causal question. The binary outcome of accepting a reverse-causality interpretation was modeled using a logistic mixed-effects model with a fixed effect of sentence type (reverse or forward causal) and random intercepts and slopes for participant and item (the maximal random effects structure). This analysis showed a significant effect of sentence type, with the reverse-causal sentences receiving more reverse-causal interpretations (ß = 3.42, p < 0.001).

In summary, this study shows that the experimental sentences from Experiments 4a–b can readily be interpreted with the last embedded clause causing the first embedded clause, and that this interpretation is even more likely with the second *that* present. The sentences are also shown to be more likely to receive the reverse-causal interpretation than sentences in which the forward-causal relationship was more sensible. This supports our contention that the rating results in Experiment 4a can be taken to bear on the availability of reverse-causal interpretations.

## 8 Additional studies

In addition to the five studies discussed above, we also conducted four other studies that showed mixed results. We report the results from these pilots and replications in the interest of full disclosure about the prospects of replication, as it seems disingenuous to report only our positive results. In two smaller rating studies similar to Experiment 1, the first showed no effect of the complementizer (ß = 0.22, p = 0.30) and the second showed a significant effect in the predicted direction (ß = 1.05, p = 0.03). The first study (n = 40) used twenty-two ambiguous sentences like in Experiment 1, including sentences 2–15 in Appendix A and eight others. The second study (n = 22) used the same materials but showed the complementizer in boldface, to test whether making the complementizer’s presence more noticeable increased comprehender sensitivity to it. Both of these pilot studies presented all sentences, without fillers, in one large table, which may have allowed participants to compare items to each other and hence made the task itself more meta-linguistic. A third study (n = 42), intended as a simple replication of Experiment 1 with a slightly modified set of stimuli (see Appendix B), failed to show an effect of the complementizer on causality judgments. The data trended in the predicted direction but complementizer presence was not a significant predictor of ratings (ß = 0.16, p = 0.19). This set of studies, then, suggests that the effect of a complementizer on causal inferences is subtle and can be quite sensitive to small changes in procedure.

Lastly, one other experiment was conducted that used a more indirect, less metalinguistic means of assessing causal interpretation, asking participants to consider the counter-factual: if the first embedded proposition had not occurred, would the second proposition still have occurred? If a participant interprets the first proposition as causing the second, then a claim about the first not having happened should entail that the second is no longer guaranteed to have happened either. But if the embedded clauses are interpreted non-causally, then canceling the first would have no impact on the second. Participants (n = 42) read the twenty-eight target sentences and thirty fillers from Experiment 1, including ten unambiguous (non-)causal sentences, ten temporal items and ten IC sentences. They were asked a question like (23) and rated their agreement on a rating scale (from definitely not – definitely yes).

(23) Do you think there would have been a riot if the mayor hadn’t been elected?

The results showed no effect of the optional complementizer (p = 0.32), whereas entirely unambiguous cues (*because, unrelated to*) in the filler sentences showed a strong effect (p < 0.001). This alternative measure was therefore clearly sensitive to causality, but did not detect an effect of the complementizer in the target items.

The results reported for Experiments 1 and 2, combined with the pilots and replication attempts listed here, illustrate the subtlety of the complementizer effect and its sensitivity to methodology. As noted earlier, we follow Schütze & Sprouse’s (2014) suggestion that experimental tasks like ratings on a Likert scale may not yield enough power to see small effects, whereas forced-choice studies can detect small differences but may magnify the effects. We take the forced-choice results, with their 80% vs. 20% choices in Experiment 2, for example, to indicate that the effect of a complementizer on causal inferences is real, but not that it is a huge effect. The events we described in our materials, particularly for the forward-causality studies, were designed to be not too tightly causally linked, precisely because we wanted to allow for the availability of the symmetric non-causal connection. In many contexts, comprehenders’ real-world knowledge enforces a causal reading on its own, so in order to see if the complementizer could have an effect, we needed these more loosely linked events. However, the looser connections may have made it more difficult for participants to have strong intuitions about the connections between these events. In this way, our studies required connections that simultaneously were sufficiently loose to permit multiple interpretations but were not so loose as to be incoherent. Our ability to see the effects of complementizers may have therefore depended on finding that delicate balance.

## 9 General discussion and conclusions

The studies presented here show that the presence of a complementizer preceding the second of two embedded clauses affects the perception of causal connection between the embedded clauses. Experiments 1 and 2 show that the optional complementizer in forward causality contexts helps reduce the perception that the event described in the first embedded clause caused the event described in the second. Experiments 3 and 4 show that a complementizer in reverse causality contexts improves naturalness, presumably because a weakening of a forward causal relationship is helpful in these contexts. Interestingly, Experiment 4b shows that backward causal relationships can arise between clauses joined by *and*, counter to the strongest interpretation of a claim by Bar-Lev and Palacas (1980). These results demonstrate that a word which functions as a syntactic cue to an embedded clause without contributing much semantic content can affect perceptions of causal coherence between clauses, highlighting the importance of taking surface syntactic cues into account in models of discourse coherence. The effect is not large, and is especially difficult to detect using naturalness ratings, but the fact that it affects both forward and backward causality relationships between clauses in a consistent manner leads us to believe that it is genuine.

### 9.1 Re-evaluating Bjorkman

The results of the main studies reported here provide support for some of Bjorkman’s (2010 (2013) syntactic observations. Specifically, her contention that coordination of unambiguous CPs (i.e., with the complementizer present) should reduce perceptions of forward causality compared to coordination of TPs is supported by Experiments 1 and 2. However, her papers leave the impression that CP coordination should be unambiguous: she states that the CP coordination should obligatorily convey a symmetric, non-causal interpretation. There should be interpretive ambiguity only when the coordination is structurally ambiguous between CP or TP coordination (i.e., without the complementizer). But our results do not show an effect of that strength. Rather, the presence of the second complementizer serves only to reduce the causal bias but not eliminate it entirely. Our results are a reminder that the interpretation a comprehender assigns likely reflects a probabilistic combination of syntactic cues and event-level reasoning, and models of language processing must account for probabilistic biases while still explaining how a comprehender arrives at a final interpretation of a given utterance.

Instead, the effect of a complementizer on causal interpretation is subtle enough to be elusive when targeted by simple causality ratings. The effects are strongest when participants are asked to draw a direct comparison between the two possible ways to express the sentence, as in the forced-choice paradigm. This paradigm focuses attention on the difference between the sentences (which may be similar to the effect achieved in the pilot study in which the complementizer was shown in boldface type), and involves a meta-linguistic judgment as well. This suggests that both CP and TP coordinations of clauses are consistent with causal relationships between clauses, and that there is only a weakening (not an elimination) of the causal interpretation when the CP is the only structure possible.

Nonetheless, if either CP or TP coordination is compatible with both interpretations, as our results suggest, then we still need to explain why the complementizer affects forward causal judgments at all. One possibility is a probabilistic processing approach based on the syntactic difference suggested by Bjorkman. Specifically, when the two embedded clauses are possibly contained within the same CP (without the second complementizer), they are syntactically linked more closely than when they occupy separate CPs, and one natural way of relating the linked clauses is by forming a cause-effect relationship (e.g., Sanders 2005 and causality by default). The second optional complementizer, on the other hand, separates the clauses syntactically and highlights the fact that both clauses link back to the earlier embedding verb. This highlighting of the connection to the embedding verb could correspond to a relative de-emphasizing of the connection to the first embedded clause. And it is this increased emphasis on the embedding relation, not only the specific syntax of CP coordination, that may lead to fewer participant inferences of causality and more interpretations on which the two embedded clauses report two equal but separate events. Additionally, the repetition of *that* may facilitate reactivation of the embedding verb. A follow-up experiment to substantiate this explanation could explore whether the verb is reactivated following *that* when it is present, and whether this reactivation is later and weaker without the optional complementizer.

Turning to production, one can consider our results in relation to the literature on speakers’ inclusion or omission of optional *that* in simpler sentences (e.g., Jaeger 2010). That literature has found that the inclusion of *that* depends on factors such as information density, the accessibility and frequency of the subject of the complement clause, and the amount of material between the matrix verb and the complement clause. In simpler sentences with only a single embedded clause, more predictable or accessible complement clauses are less likely to be marked by *that*. Our own findings can be interpreted analogously in that a pair of embedded clauses which are more tightly linked to each other (i.e., causally connected) are less likely to be marked by *that* on the second conjunct. If causality and complementizers do interact in comprehension in the ways demonstrated here, one prediction is that a manipulation of causality ought to affect speakers’ production preferences as well. Our results suggest that a speaker’s intention to convey a causal connection could act, along with other factors listed above, to decrease production of the complementizer *that* before the second conjoined clause.

### 9.2 Alternative analyses and predictions

Another possible explanation for our results is an iconic one, whereby parallelism in surface form cues symmetric (i.e., non-causal) interpretation. That is, when both embedded clauses are introduced with a complementizer, they are introduced with the same overt material. When the second complementizer is omitted, then there is an asymmetry in how the two clauses are introduced, and this asymmetry may perhaps be mapped iconically onto an asymmetric (i.e., causal) interpretation. This explanation also makes an explicit prediction: omitting a complementizer before the first embedded clause but including a complementizer before the second creates an asymmetry that should favor asymmetric (i.e. causal) interpretations. If the mechanism for the complementizer effect depends on the parallelism between the two embedded clauses, then the omission of the first complementizer but the inclusion of the second, as in (24), should increase causal interpretation.

(24) The newspaper reported the mayor was elected and that there was a riot.

Unfortunately, testing this prediction is difficult because removing the first complementizer but including the second may be dispreferred, particularly if a verb like *report* favors an overt *that* (though cf. Staum & Sag 2007 on multiple *that* as a facilitator of another construction). Any effect of the complementizer on perceived causality in (24) may be obscured by this fact. We would be inclined to mark the sentence as ungrammatical, but this is an empirical question.

Under an iconicity account, a more compact expression ought to favor a smaller conceptual distance between the components of that expression, in our case yielding the closer causal link between events mentioned in two embedded clauses when the second complementizer is omitted. Haiman (1985) discusses this link between form and meaning. He observes that linguistic formulations that put constituents closer together also yield more closely linked interpretations. Under a rational principle of least effort or economy, closely linked concepts yield smaller expressions. One example in this vein comes from the language Warekena, which expresses an “inalienable” relationship with fewer markers than an alienable one: Aikhenvald (2012) reports that Warekena uses a prefix to express inalienable possession (e.g., *your voice*), whereas a combination of a pronominal prefix and a possessive suffix are required for alienable possession (e.g., *your canoe*). In this way, linguistic reduction favors conceptual closeness whereas linguistic expansion favors conceptual distance. The iconic account makes another prediction: if another element besides the complementizer could be inserted before the second conjoined clause without its semantics favoring a particular coherence relation, this element should also reduce forward causal connections between the clauses. In auditory experiments, the presence or size of a prosodic boundary between two conjoined embedded clauses should similarly reduce forward causal inferences.

Lastly, we return to the role of implicature in deriving causal readings in these contexts. A pragmatic account posits that the semantic meaning of *and* as logical conjunction undergoes pragmatic enrichment by way of Grice’s (1975) maxim of Manner. If cooperative speakers are expected to “be orderly” in their presentation of temporally sequenced events, then listeners can rightly infer that an event described in the first conjunct likely preceded an event described in the second conjunct. The maxim of Manner likewise imposes an expectation for brevity. This underlies the observation that unmarked, briefer forms are used to convey unmarked, stereotypical meanings, whereas periphrastic or less lexicalized expressions are used to convey marked situations (Horn 2004). For example, using the longer expression *cause to die* can implicate that a death was brought about by non-stereotypical means, for which the briefer form *kill* would have been inappropriate (see Shibatani 1975; McCawley 1978; Comrie 1985). If this approach is extended to complementizers in embedded contexts, the inclusion of the complementizer creates a more prolix form, and if prolixity is associated with markedness, the use of the complementizer may in turn signal a more marked reading. If causality is the default, then the marked form that includes the optional complementizer should favor the non-causal reading.^4^

The meanings that arise for *kill* and *cause to die* seem to stand in starker contrast to each other than do the subtle variations in causal connectedness we observe. A speaker flouts the maxim of Manner in producing *cause to die* and generates a strong implicature. However, there are other contexts in which quite subtle distinctions in meaning are attributed to the calculation of implicatures via principles of cooperative communication. For example, Levinson (2000: 148–149) compares (25a, b), which vary only in the presence/absence of the second subject.

(25)
  - He went to the store and bought some whiskey.
  - He went to the store and he bought some whiskey.

Levinson observes that (25a) supports an interpretation of a single complex action of “store-going-in-order-to-whiskey-buy”, whereas (25b) more easily permits the interpretation of two independently initiated actions. This distinction is claimed to arise via a maxim of minimization: “Say as little as necessary; that is, produce the minimal linguistic information sufficient to achieve your communicational ends” (Levinson 2000: 114). In this way, a component of the maxim of Manner (minimization of form) has been invoked to explain a subtle distinction in meaning between the portrayal of a single complex event and two independent events. Examples (25a–b) are particularly analogous to our results because they manipulate the presence/absence of an optional word which serves primarily to establish structure (verb phrase coordination in (25a) versus sentence coordination in (25b)) without changing the event-level semantics of who did what to whom. As with our complementizer manipulation, the manipulation in (25a–b) achieves a change in the perceived closeness of the two actions whereby the shorter version is more tightly integrated.

In sum, our results are broadly compatible with accounts that depend in part on syntactic mechanisms, those that appeal to notions of iconicity or cooperative communication, or processing accounts which invoke information-theoretic constraints. Further empirical work will be needed to tease apart these explanations, including the follow-up ideas discussed above. What the theories do have in common is the incorporation of multiple factors in processing and an iconic interpretation of distance between clauses. We believe that the potential syntactic difference between CP and TP coordination is not the complete explanation for the processing effect, since on that theory at least one version of the structure should be unambiguous. A combination of iconic distance and parallelism of structure may provide the most compelling account. In all cases, though, the theoretical machinery involved must permit a semantically bland complementizer to have repercussions for discourse-level interpretation. In the end, that is the aspect of our findings we find most interesting—that the inference of causal coherence relations is influenced by linguistic elements that have nothing to do with causal reasoning. Such influence can be observed only by considering contexts in which coherence relations operate within the sentence, where structural forces can be brought to bear on pragmatic inferences.

## Supplementary Material

appendices

## Figures and Tables

**Table 1:** Results of Experiment 2.

Which of the two sentences below is more likely to mean that…	the student teacher not being confident caused the students’ poor behavior?	the student teacher not being confident and the students’ poor behavior were unrelated?
complementizer absent	409 (79%)	106 (20%)
complementizer present	109 (21%)	412 (80%)

**Table 2:** Mean naturalness ratings for items in Experiment 3.

	Forward causality	Reverse causality
Without *that*	5.06	4.22
With *that*	5.16	4.41

**Table 3:** Participants’ selections in forward and reverse causality contexts in Experiment 4a.

	Forward causality	Reverse causality
Without *that*	123 (40%)	51 (16%)
With *that*	186 (60%)	259 (84%)

## Footnotes

1 There is an alternative syntactic structure for (2a) in which the second clause is not embedded at all and therefore it is the report of the election that may be interpreted as causing the riot. In that case, there is only one embedded clause, and the clause about the riot is conjoined at the higher sentence level. We will return to this in the presentation of our study materials, but what is of primary interest here is the reading in which the newspaper reports two events.

2 Note that in stand-alone sentences, the forward causality and reverse causality cases are both possible, as in (i) and (ii). Illegal driving can result in police action (the inference for (i)) or it can be mentioned as an explanation (the inference for (ii)).

- Clive ran a red light. The policeman pulled him over.
- The policeman pulled Clive over. Clive ran a red light.

It is only when the two clauses are *and*-conjoined that the reverse causality reading is awkward (Bar-Lev & Palacas 1980).

3 The number of participants is large because entire classrooms of students participated. Once professors had agreed to allow their students to do the study, questionnaires were provided for all of the students.

4 That said, a quick search of the parsed *Wall Street Journal* text in the Penn Tree Bank (using tgrep2; D. L. T. Rohde 2004) found 80 cases of a “verb that S and that S” structure but only 31 of a “verb that S and S”. Further work is needed to determine if this distribution is general and what coherence relationships are expressed by each structure in corpora, but our markedness claim must be speculative at this time.

## Abbreviations

cp: Complementizer Phrase
cuny: City University of New York
ic: Implicit Causality
irb: Internal Review Board
lmer: linear mixed effects regression
msu: Morehead State University
nih: National Institutes of Health
pdtb: Penn Discourse Tree Bank
tp: Tense Phrase
