# Supplementary material for: Form and function: Optional complementizers reduce causal inferences
Source: Glossa. Author manuscript; Available in PMC 2017 Aug 10. (PMC5552188; doi:10.5334/gjgl.134)
Supplement: appendices [file NIHMS880891-supplement-appendices.pdf]

Appendix A: Experiment 1 stimuli

1. The newspaper reported that the mayor was elected and (that) there was a riot.

2. In his weekly email, the university president said that the controversial basketball coach had been fired and (that) there was new graffiti on campus.

3. At the morning assembly, the school principal announced that there was an explosion in the chemistry lab and (that) there was water shooting from a leaking pipe in the hall.

4. At the board meeting, the CEO revealed that the profits were down and (that) all employees were getting an extra week of vacation.

5. In the lecture, the professor explained that the exam date was moved back and (that) there were no extra credit opportunities.

6. In the clothing store, the saleswoman remarked that the jackets were on sale and (that) the employees were overworked.

7. During the negotiations, the defense lawyer observed that the judge was sleeping and (that) the bailiff walked out of the room.

8. In math class, the teacher remarked that half the class failed the test and (that) there was a mutiny.

9. During the interview, the politician claimed that not all votes were counted and (that) the wrong man won.

10. At the construction site, the architect estimated that the concrete was poorly prepared and (that) the building was unsafe.

11. After the wedding, the groom feared that his brother lost his car keys and (that) his dad missed his flight.

12. After school, the students understood that the buses were late and (that) everyone would get home a little late.

13. Over the phone, the police office confirmed that there was a marathon happening and (that) the streets were closed.

14. On its website, the restaurant stated that it had a new chef and (that) its dinner prices had gone down.

15. In the email, the student read that the police were offering free CPR training and (that) there was a drop in youth mortality in the state.

16. During the class observation, the professor noted that the student teacher talked quietly and (that) the students were poorly behaved.

17. Over the weekend, the business journal reported that the new superhero movie earned millions of dollars and (that) traffic was worse.

18. During his shift, the janitor assumed that the secretary worked hard and (that) her room didn't need to be cleaned.

19. While on the air, the news anchor pointed out that more people are biking to work and (that) there was a drop in air pollution.

20. While processing a request, the bank teller confirmed that the bank was starting an investing operation and (that) employees were working overtime.

21. In a call to the office, the camp workers noted that the plumbing wasn't working and (that) the kids swimming went everyday.

22. During class observation, the professor noted that the student teacher did not look confident and (that) the students were poorly behaved.

23. Over the weekend, the business journal reported that the new movie earned millions of dollars and (that) the director went on vacation.

24. At night, the janitor assumed that the secretary was working hard and (that) her room didn't need to be cleaned.

25. While on the air, the news anchor pointed out that more people are biking to work and (that) health insurance costs are continuing to rise.

26. While processing a request, the bank teller confirmed that the bank was starting an investing operation and (that) overdraft fees would be waived for the year.

27. In a call to the office, the camp workers noted that the plumbing wasn't working and (that) the kids went swimming everyday.

28. In a short article, the newspaper editor stated that the country's immigration policy had changed and (that) food prices had increased.

29. In an email, the politician declared that the state's finances were in trouble and (that) the bridge project is canceled.

30. In a press release, the video game company announced that they launched a new project and (that) there are positions available for qualified programmers.

31. In its latest post, the blogger mentioned that a new local band gave a concert and (that) there was a power outage.

32. In the courtroom, the witness testified that the defendant committed the murder and (that) the school started a new public safety campaign.

Appendix B: Experiment 2 stimuli

1. On the front page, the newspaper reported that the mayor was elected and (that) there was a riot.

2. In his weekly email, the university president said that the controversial basketball coach had been fired and (that) there was new graffiti on campus.

3. In his morning address, the school principal announced that there was an explosion in the chemistry lab and (that) there was water shooting from a leaking pipe in the hall.

4. At the board meeting, the CEO revealed that the profits were down and (that) all employees were getting an extra week of vacation.

5. In the lecture, the professor explained that the exam date was moved back and (that) there were no extra credit opportunities.

6. In a talk with the head office, the saleswoman remarked that the jackets were on sale and (that) the employees were overworked.

7. While complaining about the trial, the defense lawyer observed that the judge was sleeping and (that) the bailiff walked out of the room.

8. In the staff lounge, the teacher remarked that half the class failed the test and (that) there was a mutiny.

9. During the interview, the politician claimed that not all votes were counted and (that) the wrong man won.

10. In a memo, the architect claimed that the concrete was poorly prepared and (that) the building was unsafe.

11. While lying awake, the groom feared that his brother lost his car keys and (that) his dad missed his flight.

12. After school, the students understood that the buses were late and (that) everyone would get home a little late.

13. Over the phone, the police officer confirmed that there was a marathon happening and (that) the streets were closed.

14. On its website, the restaurant stated that it had a new chef and (that) its dinner prices went down.

15. In the email, the student read that the police were offering free CPR training and (that) there was a drop in youth mortality in the state.

16. While watching videotape of the class, the professor noted that the student teacher talked quietly and (that) the students were poorly behaved.

17. Over the weekend, the business journal reported that the new superhero movie earned millions of dollars and (that) traffic was worse.

18. While on the air, the news anchor pointed out that more people are biking to work and (that) there was a drop in air pollution.

19. In their weekly report, the camp workers noted that the plumbing wasn't working and (that) the kids went swimming every day.

20. While reading the newspaper, Jeremy learned that his doctor won the lottery and (that) the office building downtown was up for rent.

21. At night, the janitor assumed that the secretary was working hard and (that) her room didn't need to be cleaned.

22. While on the radio, the caller insisted that she did yoga last week and (that) her shoulder hurts.

23. While processing a request, the bank teller confirmed that the bank was starting an investing operation and (that) overdraft fees would be waived for the year.

24. In a short article, the newspaper editor stated that the country's immigration policy changed and (that) food prices increased.

25. In an email, the politician declared that the state's finances were in trouble and (that) the bridge project was canceled.

26. In a press release, the video game company announced that they launched a new project and (that) there are positions available for qualified programmers.

27. In its latest post, the blogger mentioned that a new local band gave a concert and (that) there was a power outage.

28. In the courtroom, the witness testified that the defendant committed the murder and (that) the school started a new public safety campaign.

Appendix C: Unambiguous causal and non-causal fillers (Experiment 1, Experiment 2)

Causal fillers

1. In the hallway, the janitor Joe Sherman congratulated Sue Nichols because she won the recent Nobel prize.

2. At the community swimming pool, the lifeguard Kyle reprimanded Sally because she was biting someone.

3. At the local French restaurant, a regular patron Mary complimented the chef Jim Clark because he recently won a cooking award.

4. At the children's literature award ceremony, Judge Frank Nelson congratulated Alice Jones because she won the Newberry Award.

5. After the soccer game, Coach Frank congratulated Alexa because she scored the winning goal.

6. In the 4th grade classroom, Mrs. Smith rebuked the boy Jim because he was farting.

7. Before the show, the conductor Mario admonished the musicians because they were performing poorly.

8. At the parade, police officer George Stone smiled at the waitress named Gloria because she was yelling.

9. In the gym, the trainer Jane reprimanded Max the bodybuilder because he was doping.

10. Backstage on opening night, the director Geoffrey criticized the actresses because they were unprepared.

Non-Causal fillers

1. In the hallway, the janitor Joe Sherman congratulated Sue Nichols unrelated to her having recently won the Nobel prize.

2. At the community swimming pool, the lifeguard Kyle reprimanded Sally unrelated to her biting someone.

3. At the local French restaurant, a regular patron Mary complimented the chef Jim Clark despite his having recently won a cooking award.

4. At the children's literature award ceremony, Judge Frank Nelson congratulated Alice Jones despite her winning the Newberry Award.

5. After the soccer game, Coach Frank congratulated Alexa despite her scoring the winning goal.

6. In the 4th grade classroom, Mrs. Smith rebuked the boy Jim unrelated to the fact that he was farting.

7. Before the show, the conductor Mario admonished the musicians despite them performing poorly.

8. At the parade, police officer George Stone smiled at the waitress named Gloria despite her yelling.

9. In the gym, the trainer Jane reprimanded Max the bodybuilder despite his doping.

10. Backstage on opening night, the director Geoffrey criticized the actresses despite them being unprepared.

Appendix D: IC vs. non-IC fillers (Experiment 1, Experiment 2)

First alternative is the IC variant; second is the non-IC variant.

1. While working out, the cyclist Patrick faulted/followed his distracted trainer Sarah.

2. During the transatlantic flight, pilot Juan Hernandez scolded/chatted_with Rosa the napping stewardess.

3. Before the test, the student James admired/waved_at his hard-working friend Amelia.

4. After the election, Congresswoman Wyshaw applauded/greeted the winning candidate Josh Henderson.

5. Before the coffee shop opened, the baker Ronald admonished/had_a_latte_with the late-arriving barista, Julia.

6. In the laboratory, the scientist Gerald Jones praised/ate_lunch_with the recently promoted technician Joanne.

7. At the art gallery opening, the curator Lucy complimented/passed_a_drink_to the prize-winning artist Horatio Smith.

8. At the local garage, truck driver Jonas Flick condemned/met the snoring mechanic Anita Lyon.

9. During a business conference, the manager Sheila lectured/introduced the texting intern Kevin.

10. When the filming ended, director Cyril Brinkmeyer honored/waved_at the retiring star Silvia Sun.

Appendix E: Forward and reverse causality sentences (Experiment 3, Experiment 4)

Forward-causality

1. The witness said that Clive ran a red light and (that) the policeman pulled Clive over.

2. Another classmate said that the teacher didn't like John's paper and (that) John ripped his paper up.

3. The janitor noticed that Sarah drank the soda and (that) she threw the can away.

4. The babysitter noticed that Billy put the cake in the oven and (that) it turned golden-brown.

5. My cousin reported that Jenny pushed Bill and (that) Bill fell off a cliff.

6. The attorney reported that Paul harassed David's wife and (that) David assassinated Paul.

7. The cheerleading coach stated that Mary dropped Rachel and (that) Rachel broke her collarbone.

8. The cook declared that Sarah knitted the sweater and (that) Greg threw it in the fire.

9. The doctor wrote that the athlete made his longest jump and (that) he broke his neck.

10. The email said that Steve burned the book and (that) the book turned to ash.

11. The hall monitor announced that the bully tripped Miles and (that) he skinned his knee.

12. The host announced that Steve knocked the egg off the counter and (that) it cracked.

13. The journalist said that Phoebe trained Mark and (that) he completed the marathon.

14. The maid said that Anne dropped the vase and (that) the vase shattered.

15. The neighbor reported that James raised the cow and (that) Bob slaughtered it.

16. The newspaper reported that Sheila suffocated Buffalo Bill and (that) he died.

17. The report said that John bombed the building and (that) the building collapsed.

18. The speaker said that Kelly won a Nobel prize and (that) Frank congratulated Kelly.

19. The story goes that the crew built the boat and (that) they set sail.

20. The bank teller reported that Mike got his first bank account and (that) he deposited some money.

Reverse-causality

1. The witness said that the policeman pulled Clive over and (that) Clive ran a red light.

2. Another classmate said that John ripped his paper up and (that) the teacher didn't like John's paper.

3. The janitor noticed that Sarah threw the can away and (that) she drank the soda.

4. The babysitter noticed that the cake turned golden-brown and (that) Billy put it in the oven.

5. My cousin reported that Bill fell off a cliff and (that) Jenny pushed Bill.

6. The attorney reported that David assassinated Paul and (that) Paul harassed David's wife.

7. The cheerleading coach stated that Rachel broke her collarbone and (that) Mary dropped her.

8. The cook declared that Greg threw the sweater in the fire and (that) Sarah knitted it.

9. The doctor wrote that the athlete broke his neck and (that) he made his longest jump.

10. The email said that the book turned to ash and (that) Steve burned the book.

11. The hall monitor announced that Miles skinned his knee and (that) the bully tripped him.

12. The host announced that the egg cracked and (that) Steve knocked it off the counter.

13. The journalist said that Mark completed the marathon and (that) Phoebe trained him.

14. The maid said that the vase shattered and (that) Anne dropped the vase.

15. The neighbor reported that Bob slaughtered the cow and (that) James raised it.

16. The newspaper reported that Buffalo Bill died and (that) Sheila suffocated him.

17. The report said that the building collapsed and (that) John bombed the building.

18. The speaker said that Frank congratulated Kelly and (that) Kelly won a Nobel prize.

19. The story goes that the crew set sail and (that) they built the boat.

20. The bank teller reported that Mike deposited some money and (that) he got his first bank account.
